# Gender Role Mindset and Beliefs about Own Personal Goals as a Guide for Young People’s Behaviors towards the Romantic Partner

**DOI:** 10.3390/bs14090818

**Published:** 2024-09-14

**Authors:** Gaia Cuccì, Camilla Chiara Colombo, Emanuela Confalonieri

**Affiliations:** CRIdee, Department of Psychology, Università Cattolica del Sacro Cuore, 20123 Milano, Italy; camillachiara.colombo@unicatt.it (C.C.C.); emanuela.confalonier@unicatt.it (E.C.)

**Keywords:** dating violence, gender stereotypes, personal values, young people

## Abstract

Dating violence (DV) is a form of intentional abuse carried out in young couples, which over the years has increasingly gained attention for its pervasiveness and high frequency. The present study represents an effort to expand and deepen the literature on factors associated with DV perpetration. The sample consisted of 225 Italian young people who completed an online survey. A model was tested, in which DV perpetration is affected by the presence of gender stereotypes and personal values oriented to power and dominance (i.e., self-enhancement) and to universalism and interest in others (i.e., self-transcendence) through the mediation of the perception of the romantic relationship quality, controlling for age. Sex was also considered in the model. The findings showed that self-enhancement and self-transcendence were, respectively, linked positively and negatively to a negative perception of the relationship quality, which in turn explained DV perpetration. The results also suggested that hostile sexism, self-enhancement and being male were directly linked to DV perpetration, thus representing potential risk factors for it. The study suggests the importance of working on gender role beliefs and personal orientation to power, which guide the way people perceive their romantic relationships and behave toward their partners, providing interesting insights for the implementation of DV prevention programs. Moreover, the findings highlight the importance of working with families and other educational agencies to foster a change in cultural terms.

## 1. Introduction

Romantic relationships play a central role in youth development [1]. The development and maintenance of intimate relationships represent, indeed, one of the major developmental tasks in both adolescence and young adulthood (e.g., [2,3]), entailing significant implications for positive mental health and psychosocial adjustment (e.g., [4,5]). Besides this, romantic relationships may also be alarmingly associated with potential harmful consequences for psychological and physical well-being (e.g., [4,6,7]). In this regard, high conflict and violence, which may frequently occur in romantic relationships of poor quality, may lay the foundation for the occurrence of dating violence (DV), a form of intentional abuse that encompasses psychological, physical and sexual violence or stalking behaviors carried out by the current or previous partner in young couples [8].

In the literature, there is no univocal categorization of types of DV. Nevertheless, the majority of authors (e.g., [9,10,11]) identified four forms of DV: *psychological violence*, which involves behaviors that are intended to exert control over the partner or to emotionally cause him/her harm; *physical violence*, which refers to the use of coercion to overpower the other person; *sexual violence*, which comprises behaviors that may range from sexual insults to sexual pression and assault; and *stalking* or *threating behaviors*, namely unwanted attention that fosters feelings of fear and concern about one’s own safety.

In recent years, DV has gained significant attention due to its pervasiveness and high rates in adolescents’ and young adults’ romantic experiences. Indeed, recent meta-analyses and reviews [12,13,14] have highlighted that DV has become a common phenomenon in young people’s close relationships for both males and females. Concerning the Italian context, a recent survey conducted on a sample of 800 Italian adolescents [15] showed that 41% of adolescents have been victimized, while 30% of adolescents have perpetrated violence in their current or former romantic relationship. To our knowledge, no study has been conducted in Italy on DV prevalence among young adults.

In the literature concerning DV, sex and age have frequently been taken into account. Sex differences have been examined regarding DV, both in adolescents and young adults, and similar findings have been found in both developmental stages. More specifically, several studies [10,12,14,16] have highlighted that DV involves both males and females. However, results on sex differences (e.g., [10,12,14]) in DV prevalence are often inconsistent since rates of perpetration and victimization showed significant variability. Nevertheless, authors agreed upon the fact that males tend to perpetrate more sexual violence than their female counterparts. Research on this topic also showed that DV is positively associated with age. In other words, DV perpetration and victimization tend to increase over time, from adolescence to young adulthood, and they involve an escalation, from milder to more severe forms of violence (e.g., [17,18,19]).

Nowadays, there is a broad literature (e.g., [10,20]) regarding the potential risk factors for DV perpetration in adolescence and young adulthood. Particularly, three main categories of risk factors were identified: individual (e.g., psychological distress, substance abuse, acceptance and positive attitudes towards violence and gender stereotypes), relational (e.g., poor romantic relationship quality or unbalanced power dynamics in the couple) and contextual (e.g., peer group characteristics, family environment and neighborhood).

Among individual risk factors, gender stereotypes have been investigated during the last decade in terms of hostile sexism (i.e., an antagonistic perspective of women who are seen as attempting to subjugate men and usurp men’s power and authority) and/or benevolent sexism (i.e., an idealized view of women who are seen as fragile creatures to take care of and to be protected) in samples of both adolescents and young adults (e.g., [21,22,23,24]). In this regard, findings revealed that hostile sexism may be a predictor of DV. However, ambivalent results emerged about the relationship between DV perpetration and benevolent sexism. As a matter of fact, some studies [21,23,25] warn about hostile sexism, depicting it as the chief predictor for the enactment of DV, while others [22,24] draw attention to the risk correlated with benevolent sexism.

In terms of cultural aspects and cognitive beliefs that may drive this behavior, alongside gender stereotypes, no studies have yet investigated the role of personal values. Schwartz [26] defined values as broad, trans-situational goals that vary in importance as guiding principles in life. The crucial content aspect differentiating among values is the motivational goals they express. In particular, in this study, we will focus on self-enhancement and self-transcendence, which are at the opposite ends of a continuum. The former emphasizes the pursuit of self-interests, power and achievement, and it is opposed to the latter, which involves interests of others, universalism and benevolence. To our knowledge, no studies have explored the association between personal values and DV perpetration.

Regarding relational risk factors, some studies (e.g., [27,28,29,30]) focused on the link between romantic relationship quality and DV, in both adolescence and young adulthood, showing an association between these variables. More specifically, a study [27] reported that a poor romantic relationship quality, marked by unhealthy dynamics and behaviors (e.g., high levels of jealousy, verbal conflict and tendency to cheat) may predict involvement in physical DV in adolescence. Moreover, the perception of lower power in the couple is significantly correlated with adolescent males’ perpetration of physical violence. Similarly, a study conducted by Cuccì et al. [30] showed that adolescents, both males and females, who perceived a power imbalance within the couple tended to engage more in DV. In line with these results a study [29], conducted on a sample of young adults, highlighted a positive association between a negative-quality romantic relationship and perpetration of physical DV. In other words, a relationship characterized by attempts to control the partner’s activity and/or the presence of power imbalance, conflict and transgressive behaviors could more easily lead to the occurrence of DV within the couple.

Although romantic relationship quality has been investigated in association with DV, the literature examining the correlation between romantic relationship quality and individual variables, such as gender stereotypes and personal values, is still meager and exclusively focused on young adults. More specifically, to our knowledge, only one study [31] has examined the role played by gender stereotypes (in terms of traditional masculinity ideology) on the perceived quality of the romantic involvement in a sample of young adult and adult males, showing the existence of a negative correlation. In other words, males who present more traditional gender beliefs (e.g., avoidance of femininity, restrictive emotionality, aggressiveness and competitiveness) are more prone to experience lower levels of romantic relationship quality (i.e., trust, self-disclosure, genuineness, empathy, comfort and communication). With regard to personal values, a recent study [32] focused on their association with romantic relationship quality in a sample of young adults and adults, suggesting that higher levels of self-transcendence values (i.e., benevolence and universalism) are related to higher levels of perceived romantic relationship quality.

In light of the above considerations, it emerges that DV is a complex phenomenon, which may need to be investigated by taking into account different factors and their interplay. In particular, it seems interesting to investigate both gender stereotypes and personal values in order to deepen our knowledge of the role of some cultural aspects in explaining DV perpetration.

Therefore, the aim of the present study is to test a model in which DV perpetration is explained by self-enhancement and self-transcendence and by the presence of hostile and benevolent sexism through the mediation of adolescents’ perception of the romantic relationship quality (i.e., the perception of the presence of conflict, antagonism and punishment within the couple), controlling for age. Sex is also considered in the model. To our knowledge, there are no studies jointly examining the role of gender stereotypes, personal values and the quality of the romantic relationship in DV perpetration. Moreover, no study has been conducted in Italy considering all the above-mentioned variables. Therefore, the present study may also fill a gap in the literature.

With this purpose, two main hypotheses are formulated. Firstly, we expect that the presence of gender stereotypes will impact on the perceived quality of the romantic relationship, increasing conflict between partners, which in turn will increase DV perpetration, as previously highlighted in the literature (e.g., [27,28,29,31]).

Secondly, we hypothesize that personal values are also associated with the perceived quality of the romantic relationship and DV perpetration. In particular, higher orientation toward power and dominance will be positively linked to poor romantic relationship quality, which consequently will increase DV perpetration. Consistently, on the other side, higher orientation to universalism and interest in others may foster a positive quality of the romantic relationship, decreasing DV perpetration (e.g., [30,32,33]).

## 2. Materials and Methods

### 2.1. Participants and Procedure

Considering our objectives and the main topic of the study, we excluded those who had never been in a romantic relationship, those who were in a relationship that ended more than six months before the survey administration and those who were cohabiting. In all these cases, the participants were automatically directed to the end of the survey and were not considered in the sample.

In order to test the desired sample size for the path analysis, an a priori power analysis was conducted using G*Power3 [34] with an alpha of 0.05 to achieve a power of 0.95. A minimum sample of 153 participants was required.

Missing observations or partially completed measures were removed preliminarily (*n* = 45). The final sample consisted of 225 Italian adolescents and young adults (45.8% males; 54.2% females) aged 17–27 years (M = 20.38; SD = 3.12). Of the participants, 58.2% were high school students, 22.7% college students, 11.1% workers and 8% both students and workers. Regarding sexual orientation, 94.7% of participants in the sample described themselves as heterosexual, 2.7% as bisexual and 2.6% as homosexual. The mean duration of the romantic relationship was 23.44 months (SD = 26.22), and the majority of the sample described the relationship as very important (64.4%).

Adolescent participants were recruited in Northern Italian high schools. We firstly contacted the headmasters who voluntarily granted the participation of the school. The headmaster identified the classes that would be involved in the study, and teachers allowed the administration of the study during their classes. The study was then presented to the students in class. Those adolescents who were interested in participating were provided with a letter presenting the study and the relative aims, together with the consent form for their parents. Both parents of minors interested in participating were required to sign the consent form to allow their children’s participation. Adolescents aged 18 or older signed the consent form by themselves. Only adolescents who returned the signed consent form were involved in the research. Then, the administration of the online survey took place at school in the presence of a researcher during classes.

Young adults voluntarily decided to participate in the research. They were recruited by posting an invitation to participate in the study on social media. Those interested filled out an online consent form and then completed the online survey.

Approval for the study was obtained from the Ethical Commission of Psychology Department of the Catholic University of the Sacred Heart of Milan.

### 2.2. Measures

We asked participants to complete an online questionnaire that took about 45 min investigating the following constructs.

#### 2.2.1. Socio-Demographic Characteristics and Dating Experience

Participants firstly completed items on socio-demographic variables regarding sex, age, job, romantic relationship status, romantic relationship length and importance and sexual orientation.

#### 2.2.2. Dating Violence Perpetration

We used a shorter version of the *Conflict in Adolescent Dating Relationships Inventory (CADRI)* [35] already used in other studies with Italian adolescent participants (e.g., [9,30]). CADRI is a self-report instrument to assess multiple forms of abusive behaviors that may occur between dating partners. The shorter version of CADRI consists of 21 items (e.g., “*I threatened to end the relationship”*, “*I insulted her/him with put-downs*” and “*I kicked, hit or punched her/him*”). Response choices for each item were measured on a 4-point Likert scale (0 = never to 3 = often; recoded as 1 = never to 4 = often). The inventory is frequency-based, and it measures both DV perpetration and victimization. In the present study, we measured only DV perpetration, and we employed only four of the original five subscales. In agreement with the ethical commission, it was decided not to assess the sexual abuse scale. This was an ethical choice related to the fact that items referring to sexual abuse could possibly hurt the sensibility of participants and be excessively strong for adolescents. In this study, we calculated the total average score to obtain a total score of DV perpetration. The alpha was 0.93. CADRI is particularly suitable for adolescents; however, a study showed that CADRI can be also suitable for young adults [36]. In doing so, we decided to select for the participation in the study only young adults who had a dating or romantic relationship experience and who were not cohabiting with their romantic partner or had never cohabited before. Indeed, to increase consistency within the sample in terms of relational status, we decided to focus on the same type of romantic relationship, thus excluding cohabitation, which is not the most common among Italian young people. Moreover, cohabitation has specific characteristics and involves other types of behaviors and implications that fall under intimate partner violence (IPV) and no longer under DV [37]. Therefore, we think that CADRI items can fit well with adolescents and young adults since they describe common conflict situations in dating romantic relationships.

#### 2.2.3. Perception of the Quality of the Romantic Relationship

We used the *Network of Relationships Inventory (NRI)* [38,39] to measure adolescent perception of relationship quality. It is a scale measuring the perception of relationship quality with other individuals (romantic partner, mother, father and friends) describing both positive and negative dimensions of the romantic relationship. The items are rated on a 5-point Likert scale from 1 (never) to 5 (always). In this study, participants were asked to complete the scale with reference to their romantic partner. Two main factors can be also calculated. The first one is social support, regarding perception of positive quality in terms of, for example, satisfaction, intimacy, affection and enhancement of worth. The second main factor is negative interactions (e.g., *“How much does this person scold you for doing something you are not supposed to do?”, “How often do you and this person get mad at or get in fights with each other?”* and “*How much do you and this person get on each other’s nerves?”*), which consists of items concerning perception of negative quality in terms of perception of conflict and antagonism between partners and the subject’s perception of partner punitive attitudes towards him/her. In the present study, we used only the negative interaction factor, which had an alpha of 0.90. The variable was labeled “negative perception of the relationship quality”.

#### 2.2.4. Personal Values

The short version of the *Portrait Values Questionnaire* (*PVQ*) [40,41] adapted for the European Social Survey (ESS) was used. This version includes verbal portraits of different people. Each portrait describes a person’s goals, aspirations or wishes that point implicitly to the importance of a value. For each portrait, respondents answer on a 6-point Likert scale from 1 (not like me at all) to 6 (very much like me), so that respondents’ own values are inferred from the self-reported similarity to people described implicitly in terms of values. For the present study, we only used two subscales, each of which represents a main class of values: self-transcendence (5 items, e.g., “*It is important to him/her to listen to people who are different from him/her. Even when s/he disagrees with them, s/he still wants to understand them*”) and self-enhancement (4 items, e.g., “*It is important to him/her to be in charge and tell others what to do. S/He wants people to do what s/he says*”). Alpha scores were as follows: self-transcendence = 0.76; self-enhancement = 0.71.

#### 2.2.5. Gender Stereotypes

We used the short versions of the *Ambivalent Sexism Inventory* (ASI) [42,43]. It has 12 items, and the subject has to report their agreement or disagreement with each statement on a 0 (strongly disagree) to 5 (strongly agree) Likert scale. The ASI has two subscales. Hostile sexism (6 items, α = 0.78) is an adversarial view of gender relations in which women are perceived as controlling over men and usurping their power (e.g., “*Women seek to gain power by getting control over men*”). Benevolent sexism (6 items, α = 0.78) idealizes women depicted as pure creatures who need protection and support, but it implies that women are weak and best suited for conventional gender roles (e.g., “*Men should be willing to sacrifice their own well-being in order to provide financially for the women in their lives*”). 

#### 2.2.6. Data Analysis

Firstly, preliminary correlations among the variables were conducted using SPSS 29. Primary analyses were conducted using Amos Graphics 29 testing a path-analysis model with a mediation and analyzing both direct and indirect links. The path analysis allows us to assess the effects of a set of variables acting on a certain outcome via multiple causal pathways and to investigate direct and indirect links among the variables as well as mediated links. Therefore, this analysis fits well with our aims.

In the theoretical model, gender stereotypes and personal values were expected to explain DV perpetration, through the mediation of a negative perception of the relationship quality.

**Figure 1:**
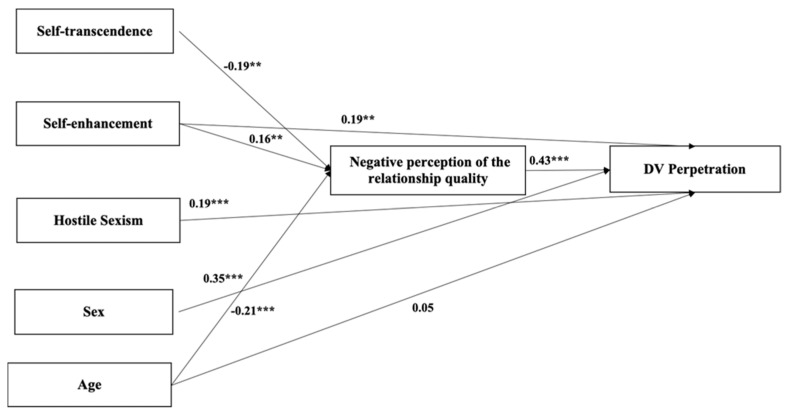
The final model. Note: ** *p* < 0.01; *** *p* < 0.001.

The model was implemented using the asymptotic distribution free estimation method, used to address the non-normal distribution of the data [44]. Starting from a saturated model with all the direct and indirect links, we then proceed with a step-by-step procedure by removing all non-significant links among variables in the model. In order to identify specific areas of the model that need adjustment, we also checked modification indices. At each step of the procedure, the goodness-of-fit indices of the model were examined considering the Chi square test, RMSEA and CFI. Models with acceptable or good fit present a Chi square > 0.05, an RMSEA < 0.08 and a CFI > 0.95 [45]. The final version of the model (Figure 1) is the result of the correlations and the estimated paths, and it only contains significant links (standardized estimates) and the evaluation of the modification indices. In the Results section, the final version of the model is reported.

## 3. Results

### 3.1. Descriptive Statistics and Preliminary Analysis

Descriptive statistics (i.e., means and standard deviations) for the considered variables and preliminary correlations are reported in Table 1.

Concerning preliminary correlations, significant associations emerged. In particular, hostile sexism was found to be positively correlated with self-transcendence (r = −0.334; *p* ≤ 0.01) and negatively correlated with self-enhancement (r = 0.138; *p* ≤ 0.05). Benevolent sexism was positively associated with hostile sexism (r = 0.317; *p* ≤ 0.01). Negative perception of the relationship quality was found to be negatively correlated with self-transcendence (r = −0.218; *p* ≤ 0.01) and positively correlated with self-enhancement (r = 0.142; *p* ≤ 0.05) and hostile sexism (r = 0.216; *p* ≤ 0.01). DV perpetration was found to be positively correlated with self-enhancement (r = 0.217; *p* ≤ 0.01), hostile sexism (r = 0.178; *p* ≤ 0.05) and negative perception of the relationship quality (r = 0.490; *p* ≤ 0.01).

### 3.2. Primary Results

As previously described, we conducted a path analysis. In the final model (Figure 1), personal values indirectly explained DV perpetration through the mediation of a negative perception of the relationship quality. In particular, self-transcendence was negatively linked to negative interactions (β = −0.192; *p* = 0.004), while self-enhancement was found to be positively linked to a negative perception of the relationship quality (β = 0.166; *p* = 0.005), which in turn was positively linked to DV perpetration (β = 0.428; *p* < 0.001). Self-enhancement was also positively and directly linked to DV perpetration (β = 0.196; *p* = 0.001). Total effects are reported in Table 2.

As for gender stereotypes, hostile sexism was found to directly and positively explain DV perpetration (β = 0.193; *p* < 0.001). A significant positive effect of sex on DV perpetration was also found, which indicated that being male is associated with DV perpetration (β = 0.351; *p* < 0.001).

As for the controlling variable, a significant effect of age emerged on the negative perception of the relationship quality (β = −0.211; *p* < 0.001), while the effect on DV perpetration was not significant.

Regarding goodness of fit, the model presented excellent indices: χ^2^_(3)_ = 3.397 (*p* = 0.334), CFI = 0.997 and RMSEA = 0.024. The final model is represented in Figure 1.

## 4. Discussion

The purpose of this study was to jointly investigate the role of gender stereotypes, personal values and the perceived quality of the romantic relationship in DV perpetration, enriching and deepening the literature on potential risk factors associated with DV. More specifically, we wanted to shed light on the role of some personal and cultural beliefs (in terms of gender stereotypes and personal values) and how they may be related to partners’ perception of the quality of the romantic relationship and to the behaviors carried out within the couple.

With regard to gender stereotypes, we found that only hostile sexism was significant in the model, while benevolent sexism showed no significant estimate paths. In particular, hostile sexism was only directly and positively linked to DV perpetration, differently from our first hypothesis. This means that those young people who have a stereotypical mindset, where women are seen as antagonists who want to dominate and control their male counterparts, are more prone to engage in DV toward their partner. This is in line with the previous literature (e.g., [21,23,25]) depicting hostile sexism as one the most significant predictors of DV. The presence of this kind of gender stereotype supports aggressive behavior since violence may become a way to behaviorally express one’s own mindset and a way to limit the partner perceived as an opponent. What seems interesting is that hostile sexism only directly impacted DV, not being related to the perceived quality of the relationship. We can hypothesize that the gender stereotype itself supports aggressive behaviors that confirm the beliefs an individual holds. Therefore, the relationship may not be perceived as positive or negative, because the conflict in the relationship is considered as “normal and acceptable”, thus confirming the individuals’ representation of the relationship. Moreover, the language referred to in hostile sexism recalls the patriarchal culture that assumes the superiority of men over women, and it is anchored to aggressive terminology. This may convey and encourage the enactment of violent behavior. As for benevolent sexism, the fact that it was not related to either the mediator or the outcome may be due to the fact that this kind of gender stereotype uses a smooth and sanitized terminology idealizing women, being depicted as weak and in need of protection. This may be more related to DV victimization (e.g., [22]), rather than DV perpetration. We can, indeed, hypothesize that benevolent sexism may be more present in victims of DV that resort to this kind of stereotype to justify the violence and/or have a mental representation of the romantic relationship as characterized by a woman that should take care of the male partner, thus confirming the stereotype. Future studies may investigate this hypothesis to enrich the understanding of the role of benevolent sexism in DV.

As far as personal values are concerned, the findings of the present study suggest that they seem to guide both the viewpoint on the relationship and the behaviors towards the partner. We think that this is particularly noteworthy since this is an uninvestigated aspect in the literature concerning DV. In line with our second hypothesis, the results showed that self-enhancement was linked to a negative perception of the relationship quality, which in turn affected DV perpetration, increasing the probability of engaging in DV. Therefore, it seems that individuals that pursue self-centered goals of power and achievement tend to develop a more negative representation of romantic relationships as characterized by antagonism and conflict between the partners. Consequently, this perception increases the engagement in DV perpetration. From this point of view, DV may represent an immature and dysfunctional attempt to relate with the romantic partner, to have power in the relationship and to manage conflicts within the couple, as suggested by previous studies [30,32,33]. Self-enhancement was also found to be directly related to DV perpetration, suggesting that a higher orientation to power and dominance over others may represent a potential risk factor that increases the frequency of DV. Again, this may be due to the fact that power is a pivotal factor when considering romantic relationships. In particular, holding the value of self-enhancement may lead a partner to gain more power in the relationship, thus eliciting conflict within the couple. The literature, indeed, showed that partners’ dissatisfaction about the level of power in the relationship, also in terms of decision-making authority, is associated with DV perpetration in young people (e.g., [30,33,46]).

On the other side, consistent with our second hypothesis, self-transcendence was indirectly linked to DV perpetration through the mediation of the negative perception of the relationship quality, reducing the probability of engaging in DV. In other words, those young people who are oriented toward others, pursuing the preservation and strengthening of others’ wellbeing as a personal goal, are less inclined to evaluate their relationship as poor in quality and conflictual, as reported in a recent study [32]. This in turn decreases the levels of DV perpetration. Considering the meager literature on the topic, we can hypothesize that being oriented toward one’s partner may help in understanding one’s own needs, values and goals, thus fostering skills (e.g., perspective-taking, empathy and mutuality) that are usually associated with positive romantic relationships [47,48]. Therefore, self-transcendence may be considered as a potential protective factor for healthier romantic relationships.

Furthermore, the quality of the romantic relationship, as expected, was confirmed as being extremely important as a mediator in explaining DV. As a matter of fact, the perception of a relationship characterized by conflict, antagonism and punitive behaviors had a strong impact on increasing the engagement in DV perpetration. In line with the literature (e.g., [27,29]), partners involved in poor-quality romantic relationships may dysfunctionally resort to violent behaviors as a strategy to solve conflicts, to control or obtain power over the partner or to express feelings of dissatisfaction. These kinds of aggressive behaviors may be expressions of immaturity or the consequences of a lack of specific skills needed to develop and maintain healthy and high-quality romantic relationships (e.g., [48,49]).

As for sex, we found that it is only directly related to DV perpetration. Therefore, males seemed to perpetrate more DV than their female counterparts. As previously mentioned, rates of DV prevalence across studies (e.g., [10,12,14]) are often inconsistent when investigating sex differences. This could be due to the use of different measures and, above all, to the types of violence that are considered.

Finally, as for the controlling variable, findings regarding age support that the quality of the relationship increases with age. From a developmental point of view, this may be due to the fact that, progressively, relationships become more stable and committed (e.g., [50]), thus requiring higher partner involvement and more sophisticated romantic skills. Age, somewhat unexpectedly, was not related to DV perpetration, as shown in previous studies (e.g., [17,18,19]). This could be because, in this sample and in the model we tested, other variables may affect the perpetration of DV more. Future studies may replicate the study and further investigate this association.

Therefore, the tested model confirms the importance of considering the individual perception of the romantic relationship quality as a pivotal factor for DV. Moreover, the findings allowed us to better understand the role of some cultural beliefs, confirming the strong influence of gender stereotypes on DV perpetration and highlighting the importance of enlarging the perspective to personal value orientation.

Although the findings provided important results enriching the knowledge of risk factors associated with DV perpetration, the present study is not without limitations. Firstly, the cross-sectional nature of the study does not allow for conclusions about causal links among the variables; thus, longitudinal designs are needed in the future. Secondly, the sample may not be representative of the entire population of Italian young people, thus raising issues with respect to the generalizability of the results. Moreover, CADRI [35] is a frequency-based measure of DV; thus, we did not evaluate motivations or other contextual variables that may affect DV perpetration. Future studies using mixed methods may provide additional clues to better understand the phenomenon of DV. Furthermore, we used CADRI for both adolescents and young adults, and it seems that CADRI can also be a useful measure to evaluate DV among the latter, since the items describe conflict situations that are also common among young adults, as suggested by Cascardi et al. [36]. However, a limitation of this measure is that it does not assess more severe forms of violence. Also, we did not administer the subscale of sexual abuse, thus not considering all the types of DV when calculating the general score. Future studies should be conducted also assessing more severe forms of DV. Moreover, we only considered as a mediator the negative perception of the romantic relationship quality, which, as expected, is a potential risk factor for DV perpetration. Future studies may also investigate the role of a positive perception of the romantic relationship in mitigating the engagement in DV perpetration, thus considering it as a potential protective factor. Finally, in the future, it could be interesting to replicate this study with a cross-cultural perspective, investigating how gender stereotypes and personal values may function differently on the basis of the culture of origin.

## 5. Conclusions

The present study provides interesting insights for the implementation of DV prevention programs. In particular, our findings suggest that working on personal beliefs and value orientation leads to a better perception of the quality of the romantic relationship, thus reducing the engagement in DV toward the partner. It is, indeed, important to make room for interventions aimed at modifying a mindset based on strong gender roles and power-oriented goals. In addition, the study highlighted that having a value orientation toward others, thus having interest in others and in their well-being as a personal goal, may be a protective factor to be empowered. At the same time, however, we believe that gender stereotypes and personal values have a strong cultural matrix and are transmitted within families or society (e.g., social media, schools, etc.). Therefore, it is also essential to work with families and other educational agencies to foster a change in cultural terms more effectively.

## Figures and Tables

**Table 1:** Means, standard deviations and correlations between variables.

	Mean (SD)	1	2	3	4	5	6
Self-transcendence	4.74 (0.80)	-					
Self-enhancement	3.51 (0.98)	0.045	-				
Hostile sexism	2.63 (0.75)	−0.334 **	0.138 *	-			
Benevolent sexism	3.01 (0.80)	−0.116	0.01	0.317 **	-		
Negative perception of the relationship quality	2.19 (0.72)	−0.218 **	0.142 *	0.216 **	0.098	-	
DV perpetration	1.63 (0.43)	−0.078	0.217 **	0.178 *	0.101	0.490 **	-

Note: * *p* < 0.05; ** *p* < 0.01.

**Table 2:** Total effects.

	DV Perpetration
Self-transcendence	−0.082
Self-enhancement	0.267

## Data Availability

The data presented in this study are available from the corresponding author upon reasonable request.
